# Acute kidney injury increases the risk of end-stage renal disease after cardiac surgery in an Asian population: a prospective cohort study

**DOI:** 10.1186/s12882-017-0476-y

**Published:** 2017-02-13

**Authors:** Sophia Tsong Huey Chew, Roderica Rui Ge Ng, Weiling Liu, Khuan Yew Chow, Lian Kah Ti

**Affiliations:** 1 Department of Anaesthesiology, Singapore General Hospital, 20 College Road, Academia, Level 5, Singapore, 169856 Singapore; 2 Department of Cardiovascular and Metabolic Disorders, Duke-National University of Singapore Graduate Medical School, 8 College Road, Singapore, 169857 Singapore; 3 Yong Loo Lin School of Medicine, National University of Singapore, 1E Kent Ridge Road, Singapore, 119228 Singapore; 4 Health Promotion Board/NRDO, 3 Hospital Avenue, Singapore, 168937 Singapore; 5 Department of Anaesthesia, National University Health System, 5 Lower Kent Ridge Road, Singapore, 119074 Singapore

**Keywords:** Acute kidney injury, Cardiac surgical procedures, Kidney failure, Chronic, Perioperative care

## Abstract

**Background:**

Acute kidney injury (AKI) after cardiac surgery is associated with increased morbidity and mortality. The long-term association between AKI and end-stage renal disease (ESRD) in an Asian population is unknown. Given the high prevalence of diabetes and a younger age of presentation for cardiac surgery, it is important to track this progression of kidney disease. Therefore, we studied the long-term risk of ESRD and mortality in our Asian patients who developed AKI after cardiac surgery.

**Methods:**

With ethics approval, we prospectively recruited 3008 patients who underwent cardiac surgery in Singapore between 2008 and 2012, and followed them up till 2014. ESRD and mortality information were obtained from the Singapore Renal Registry and Singapore Registry of Births and Deaths respectively. AKI was defined using the Acute Kidney Injury Network (AKIN) criteria, and ESRD was defined as stage 5 chronic kidney disease requiring renal replacement therapy. The Cox proportional hazards regression model was used to analyze associations between AKI and the primary outcome of ESRD and the secondary outcome of death.

**Results:**

The AKI incidence was 29.1%. During a mean follow-up of 4.4 ± 2.8 years, 0.9% developed ESRD. The hazard ratio (HR) for developing ESRD was 4.7 (95% C.I. = 1.736–12.603, *p* = 0.002) for AKIN stage 1 patients, and 5.8 (95% C.I. = 1.769–18.732, *p* = 0.004) for AKIN stage 2 and 3 patients; while the HR for mortality was 1.7 (95% C.I. = 1.165–2.571, *p* = 0.007) for AKIN stage 1 patients, and 2.5 (95% C.I. = 1.438–4.229, *p* < 0.001) for AKIN stage 2 and 3 patients.

**Conclusions:**

AKI is associated with ESRD and mortality after cardiac surgery in our Asian population. The trajectory from AKI to ESRD is rapid within 5 years of cardiac surgery. A concerted periodic follow-up assessment is advocated for AKI patients post-cardiac surgery.

## Background

Acute kidney injury (AKI), a common complication after cardiac surgery, occurs in one out of three patients [1–3]. While it has been previously thought that AKI resolves with no long-term sequelae, it is now increasingly recognized that it is associated with development of chronic kidney disease (CKD), end-stage renal disease (ESRD) and increases long-term mortality [4–9].

Amongst cardiac surgical patients, Rydén et al. demonstrated that a small increase in serum creatinine was associated with an almost 3-fold increase in the long-term risk of developing ESRD [10]; while Lo LJ et al. found that amongst all hospitalized patients, dialysis-requiring AKI patients had a 28-fold increased risk of developing stage 4 chronic kidney disease or ESRD, compared with patients who did not develop AKI [11].

This association between an episode of AKI after cardiac surgery and the risk of subsequent development of ESRD in an Asian population is unknown. It is therefore important to track the trajectory of progression of kidney disease following an acute episode of AKI in our Asian population, especially as the health and financial burden of ESRD is likely increased with our high prevalence of diabetes and younger age of presentation for cardiac surgery. Furthermore, given the extremely high local burden of ESRD [12], we embarked on this study to determine the long-term risk of ESRD and mortality in Singapore post-AKI after cardiac surgery.

## Methods

### Study population

With Institutional Review Board approval (Domain Specific Review Board 2015/00398) and written informed consent, we prospectively followed up all adult patients above 21 years old from August 2008 till 30 December 2014. These patients underwent cardiac surgery at two main heart centres in Singapore between August 2008 and July 2012. Perioperative safety and outcomes data were prospectively collected and entered into a cardiac anaesthesia database. The exclusion criteria for analysis were: (1) non-citizens and non-permanent residents; (2) patients on existing dialysis; (3) pre-existing ESRD patients; and (4) inpatient mortality cases.

### Definition of eGFR, normal serum creatinine, AKI, ESRD

The estimated glomerular filtration rate (eGFR) was calculated using the Chronic Kidney Disease Epidemiology Collaboration equation [13].

Normal serum creatinine was defined as 60 to 105 μmol/L for males and 40 to 75 μmol/L for females.

AKI was defined using the Acute Kidney Injury Network (AKIN) criteria - stage 1: ≥26.4 μmol/L or ≥50–100%; stage 2: 100–200%; stage 3: ≥354 μmol/L with an acute increase of ≥44 μmol/L or ≥200% increase between pre- and post-operative serum creatinine. The pre-operative serum creatinine was defined as the creatinine value recorded within 1 week before surgery unless there was a material change in the patient’s condition; while the peak post-operative serum creatinine was the highest creatinine within 48 h after surgery [6].

ESRD was defined as stage 5 CKD (where the glomerular filtration rate was <15 ml/min/1.73 m^2^) requiring renal replacement therapy [14].

### Outcome data

All Singaporean citizens and permanent residents have a unique personal identification number. Identification of ESRD patients amongst the cohort was accomplished through record linkage of cohort files with the Singapore Renal Registry of the National Registry of Diseases Office. This is a nationwide database of patients who have received renal replacement therapy and includes the date of entry into a renal replacement program and/or renal transplantation. The data capture for ESRD is estimated to be 95% complete [15]. This study used the date of first renal replacement therapy to define the start of ESRD as reported to the Registry.

Survival records were captured through matching of patient’s identification number with the Singapore Registry of Births and Deaths.

### Statistical analysis

Population demographics, medical history, pre-operative risk assessment, intra-operative variables and post-operative outcomes were analysed descriptively. Univariate analyses were done using 2-tailed unpaired *T*-test for continuous variables and Chi-square for categorical variables to identify the risk factors for ESRD. Patients contributed person-time in days from the time of surgery and were censored at the time of death from any cause, start of renal replacement therapy or end of the follow up period (December 30, 2014), whichever is earlier. The Cox proportional hazards regression model was used to analyse associations between AKI and the primary outcome of ESRD and the secondary outcome of death. The statistical significance of variables was taken as *p* < 0.05. All statistical analyses were performed using IBM SPSS version 22.0 (Armonk, NY, USA).

## Results

The patient characteristics are shown in Table 1. A total of 3008 patients were recruited during the study period. The total number of patients who met the inclusion and exclusion criteria was 2666. The mean age was 59.3 ± 10.5 years of which 523 were women.

**Table 1:** Univariate analysis of perioperative factors and acute kidney injury for the whole cohort

Variables		Whole cohort (*n* = 2666)	No AKI (*n* = 1889)	AKIN 1 (*n* = 608)	AKIN 2 and 3 (*n* = 169)	*p*-value
Pre-operative						
Age (years)		59.3 ± 10.5	58.0 ± 10.5	62.3 ± 9.6	62.4 ± 10.6	<0.001
Gender: male		2143 (80.4)	1534 (81.2)	486 (79.9)	123 (72.8)	0.029
Ethnicity	Chinese	1912 (71.7)	1390 (73.6)	414 (68.1)	108 (63.9)	<0.001
	Malay	425 (15.9)	271 (14.3)	126 (20.7)	28 (16.6)	
	Indian	329 (12.3)	228 (12.1)	68 (11.2)	33 (19.5)	
Body mass index (kg/m^2^)		25.0 ± 4.1	24.9 ± 4.0	25.4 ± 4.3	24.9 ± 4.7	0.032
Diabetes mellitus		1205 (45.2)	793 (42.0)	294 (48.4)	118 (69.8)	<0.001
Hypertension		1998 (74.9)	1348 (71.4)	503 (82.7)	147 (87)	<0.001
Myocardial infarction		1559 (58.5)	1072 (56.7)	379 (62.3)	108 (63.9)	0.017
Congestive cardiac failure		435 (16.3)	260 (13.8)	130 (21.4)	45 (26.6)	<0.001
Renal impairment		225 (8.4)	90 (4.8)	99 (16.3)	36 (21.3)	<0.001
Beta-blocker		1998 (74.9)	1377 (73.1)	482 (79.3)	139 (82.2)	0.001
ACE-inhibitor		1300 (48.8)	905 (48.0)	306 (50.3)	89 (52.7)	0.365
Calcium channel blocker		521 (19.5)	331 (17.6)	144 (23.7)	46 (27.2)	<0.001
Statin		2159 (81.0)	1526 (81.0)	494 (81.7)	139 (82.2)	0.881
Diuretic		480 (18.0)	276 (14.6)	149 (24.5)	55 (32.5)	<0.001
Ejection fraction (<30%)		185 (6.9)	110 (5.9)	58 (9.6)	17 (10.2)	0.002
Pre-operative creatinine (μmol/L)		91.0 ± 42.7	85.7 ± 34.6	103.5 ± 51.5	98.7 ± 35.5	<0.001
Pre-operative eGFR (ml/min/1.73 m^2^)		81.0 ± 21.6	84.8 ± 19.3	71.9 ± 23.9	71.7 ± 23.9	<0.001
Pre-operative haemoglobin (g/dL)		13.5 ± 1.7	13.8 ± 1.6	13.1 ± 1.8	12.7 ± 1.8	<0.001
EuroSCORE logistic		3.6 ± 4.6	3.1 ± 3.7	4.8 ± 6.0	4.9 ± 6.2	<0.001
Surgical variables						
Coronary artery bypass graft surgery		2191 (82.2)	1546 (82.2)	506 (83.4)	139 (82.2)	0.802
Valvular surgery		622 (23.3)	433 (22.9)	148 (24.3)	41 (24.3)	0.733
Cardiopulmonary bypass		2578 (96.7)	1820 (96.3)	595 (97.9)	163 (96.4)	0.188
IABP		249 (9.3)	130 (6.9)	89 (14.7)	30 (17.9)	<0.001
Cardiopulmonary bypass time (min)		113.5 ± 49.0	107.0 ± 43.4	125.7 ± 56.2	137.3 ± 64.7	<0.001
Aortic cross clamp time (min)		67.2 ± 36.4	63.5 ± 32.8	74.1 ± 42.5	79.5 ± 46.0	<0.001
Inotrope use		1652 (62.0)	1120 (59.9)	409 (67.7)	123 (72.8)	<0.001
Haemodilution						
Prebypass haematocrit (%)		37.8 ± 4.9	38.3 ± 4.6	36.9 ± 5.3	35.8 ± 4.8	<0.001
Lowest haematocrit during bypass (%)		24.4 ± 3.9	24.9 ± 3.9	23.4 ± 3.7	22.3 ± 3.8	<0.001
RBC transfusion		746 (28.0)	426 (22.6)	238 (39.1)	82 (48.5)	<0.001
Intensive care unit variables						
Inotrope use		1228 (46.1)	780 (41.3)	336 (55.4)	112 (66.3)	<0.001
New need for dialysis		59 (2.2)	12 (0.6)	21 (3.5)	26 (15.4)	<0.001
Lowest haemoglobin in 1st 24 h (g/dL)		9.7 ± 1.6	9.9 ± 1.5	9.2 ± 1.4	8.8 ± 1.4	<0.001
RBC transfusion		1010 (37.9)	608 (32.2)	303 (49.9)	99 (58.6)	<0.001
Total length of hospitalization (days)		11.0 ± 10.6	9.2 ± 5.6	13.2 ± 9.6	22.0 ± 30.2	<0.001
Outcomes						
End-stage renal disease		24 (0.9)	6 (0.3)	12 (2.0)	6 (3.6)	<0.001
5-year mortality		128 (4.8)	68 (3.6)	41 (6.7)	19 (11.2)	<0.001

Data represent number (percent) or mean ± standard deviation

*AKI* Acute Kidney Injury, *AKIN* Acute Kidney Injury Network, *EuroSCORE* European System for Cardiac Operative Risk Evaluation, *eGFR* estimated glomerular filtration rate, *IABP* intra-aortic balloon pump, *RBC* red blood cell

### Overall incidence of AKI and ESRD

The overall incidence of post-operative AKI in our cohort was 29.1% (*n* = 777 out of 2666). Of these AKI patients, 78% (*n* = 608), 17.5% (*n* = 134), and 4.5% (*n* = 35) developed AKI of AKIN stage 1, 2 and 3 respectively.

72.7% (*n* = 1938 out of 2666) of patients had normal pre-operative serum creatinine. The mean pre-operative eGFR for the cohort was 81.0 ± 21.6 mL/min/1.73 m^2^. Patients who developed AKI were males, older, had diabetes mellitus and hypertension, and pre-operative anaemia, poorer pre-operative renal function and lower nadir haematocrit during bypass. A total of 59 (2.2%) patients had a new need for dialysis in the post-operative period (Table 1).

During a mean follow-up of 4.4 ± 2.8 years, a total of 24 patients (0.9%) developed ESRD, including 12 (out of 608, 2.0%) with AKIN stage 1 AKI, 5 (out of 134, 3.7%) with AKIN stage 2 AKI, and 1 (out of 35, 2.9%) with AKIN stage 3 AKI. 6 patients (out of 1889, 0.3%) without AKI developed ESRD within the follow-up period. Overall, patients who developed AKI had a 7.4 times (95% Confidence Interval [C.I.] 2.943–18.822) significant higher risk of developing ESRD (*p* < 0.001), compared with patients who did not develop AKI.

The mean age of patients at the diagnosis of ESRD was 57.3 ± 9.4 years. 87.5% of the ESRD patients were males. Patients who developed ESRD had a poorer pre-operative renal function, lower pre-operative haemoglobin and lower nadir haematocrit during bypass, than those who did not develop ESRD (Table 2).

**Table 2:** Univariate analysis of perioperative factors and end-stage renal disease for the whole cohort

Variables		ESRD (*n* = 24)	No ESRD (*n* = 2642)	*p*-value
Pre-operative				
Age (years)		57.3 ± 9.4	59.3 ± 10.5	0.604
Gender: male		21 (87.5)	2122 (80.3)	0.345
Ethnicity	Chinese	14 (58.3)	1898 (71.8)	0.304
	Malay	5 (20.8)	420 (15.9)	
	Indian	5 (20.8)	324 (12.3)	
Body mass index (kg/m^2^)		25.2 ± 5.1	25.0 ± 4.1	0.839
Diabetes mellitus		23 (95.8)	1182 (44.7)	<0.001
Hypertension		22 (91.7)	1976 (74.8)	0.058
Myocardial infarction		18 (75.0)	1541 (58.3)	0.099
Congestive cardiac failure		8 (33.3)	427 (16.2)	0.045
Renal impairment		15 (62.5)	210 (7.9)	<0.001
Beta-blocker		21 (87.5)	1977 (75.0)	0.158
ACE-inhibitor		12 (50)	1288 (48.8)	0.910
Calcium channel blocker		10 (41.7)	511 (19.4)	0.016
Statin		24 (100.0)	2135 (81.1)	0.014
Diuretic		8 (33.3)	472 (17.9)	0.061
Ejection fraction (<30%)		22 (91.7)	2440 (93.0)	0.683
Pre-operative creatinine (μmol/L)		235 ± 127	89 ± 36	<0.001
Pre-operative eGFR (ml/min/1.73 m^2^)		36.3 ± 25.1	81.4 ± 21.1	<0.001
Pre-operative haemoglobin (g/dL)		11.2 ± 1.7	13.6 ± 1.7	<0.001
EuroSCORE logistic		6.9 ± 9.0	3.5 ± 4.5	0.086
Surgical variables				
IABP		4 (16.7)	245 (9.3)	0.276
Cardiopulmonary bypass time (min)		103 ± 43	113 ± 49	0.325
Aortic cross clamp time (min)		61 ± 25	67 ± 37	0.424
Inotrope use		21 (87.5)	1631 (62.3)	0.011
Hemodilution				
Prebypass haematocrit (%)		31.5 ± 4.9	37.9 ± 4.8	<0.001
Lowest hematocrit during bypass (%)		21.5 ± 3.1	24.4 ± 4.0	<0.001
RBC transfusion		13 (54.2)	733 (27.7)	0.004
Intensive care variables				
Inotrope use		13 (54.2)	1215 (46.0)	0.426
New need for dialysis		5 (20.8)	54 (2.0)	<0.001
Lowest haemoglobin in 1st 24 h (g/dL)		8.4 ± 1.4	9.7 ± 1.6	<0.001
RBC transfusion		15 (62.5)	995 (37.7)	0.013
Total length of hospitalization (days)		14.7 ± 10.5	10.9 ± 10.6	0.083
Outcomes				
Acute kidney injury		18 (75.0)	759 (28.7)	<0.001
5-year mortality		6 (25.0)	122 (4.6)	0.001

Data represent number (percent) or mean ± standard deviation

*ESRD* end-stage renal disease, *EuroSCORE* European System for Cardiac Operative Risk Evaluation, *eGFR* estimated glomerular filtration rate, *IABP* intra-aortic balloon pump, *RBC* red blood cell

**Fig. 1:**
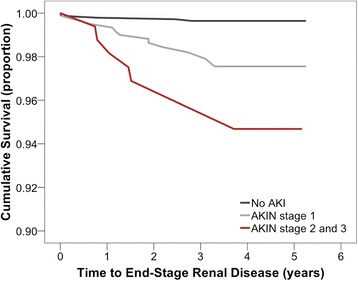
Time to end-stage renal disease for the whole cohort

The incidence of ESRD increased with advancing AKIN stage (Fig. 1). Compared with the non-AKI patients, the risk of developing ESRD was 6 and 11 times higher for patients who developed AKI of AKIN stage 1, and stage 2 and 3 respectively (*p* < 0.001, *p* < 0.001) (Table 2). After multivariate Cox regression analysis, the hazard ratio (HR) for developing ESRD was 4.7 (*p* = 0.002) for AKIN stage 1 AKI patients, and 5.8 (*p* = 0.004) for AKIN stage 2 and 3 AKI patients. Other independent risk factors in the multivariate analysis include diabetes mellitus (HR = 20.607, *p* = 0.003) and lowest haematocrit during bypass (HR = 0.900, *p* = 0.032) (Table 3).

**Table 3:** Cox regression analysis of perioperative factors and end-stage renal disease and 5-year all-cause mortality

Variables	End-stage renal disease			5-year all cause mortality		
	HR	95% C.I.	*p*-value	HR	95% C.I.	*p*-value
Diabetes mellitus	20.6	2.764–153.653	0.003	1.7	1.168–2.438	0.005
Lowest haematocrit during bypass (%)	0.9	0.818–0.991	0.032	0.9	0.901–0.988	0.013
AKIN stage 1 AKI	4.7	1.736–12.603	0.002	1.7	1.165–2.571	0.007
AKIN stage 2–3 AKI	5.8	1.769–18.732	0.004	2.5	1.438–4.229	<0.001

*AKIN* Acute Kidney Injury Network, *C.I.* Confidence Interval, *HR* Hazard Ratio

### All-cause 5-year mortality

During the follow up period, 95% (*n* = 2538 out of 2666) of patients were alive. The 5-year mortality was 4.8% (*n* = 128 out of 2666).

The mortality amongst the AKI patients was significantly higher than non-AKI patients (7.7 vs 3.6%, *p* < 0.001). 6.7% of patients with AKI of AKIN stage 1 (*n* = 41 out of 608) died within 5 years of surgery, whereas 10.4 and 14.0% of patients with AKI of AKIN stage 2 (*n* = 14 out of 134) and 3 (*n* = 5 out of 35) died within 5 years of surgery respectively (Table 1).

**Fig. 2:**
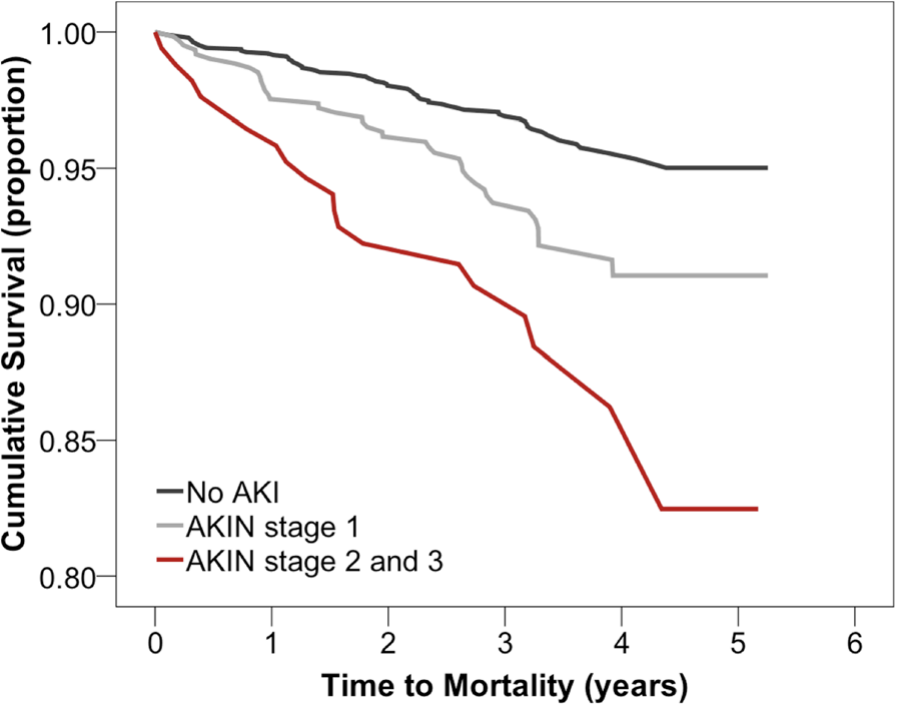
Time to mortality for the whole cohort

There was a significant dose-dependent relationship between the severity of AKI and mortality (*p* < 0.001) (Fig. 2). The risk of mortality was 2 and 3 times significantly higher for patients who developed AKI of AKIN stage 1, and stage 2 and 3 respectively (*p* = 0.002, *p* = 0.012) (Table 2). The HR for mortality was 1.7 (*p* = 0.007) for AKIN stage 1 AKI patients, and 2.5 (*p* < 0.001) for AKIN stage 2 and 3 AKI patients (Table 3).

Patients who developed ESRD had a 5.4 times (95% C.I. = 2.650–11.059) significant higher risk of developing all-cause 5-year mortality compared to patients who did not develop ESRD (25.0 vs 4.6%, *p* = 0.001).

### Relationship between eGFR, AKI and ESRD

There is an inverse relationship between eGFR and ESRD. 88.9% of patients who developed ESRD have eGFR <60 ml/min/1.73 m^2^ as compared to 5.6% of patients with normal eGFR (≥90 ml/min/1.73 m^2^) and 5.6% with eGFR between 60 and <90 ml/min/1.73 m^2^ (*p* < 0.001).

2.3% (18 out of 777) of patients with AKI developed ESRD as compared to 0.3% (6 out of 1889) of patients who did not have AKI but developed ESRD (*p* < 0.001).

## Discussion

AKI is a serious complication with adverse long-term outcomes. Hitherto, AKI was believed to be self-limiting [8] but within a 4.4 ± 2.8 years follow-up period, 0.9% patients locally who underwent cardiac surgery developed ESRD.

There is a dose-dependent relationship between AKI and the risk of ESRD. Patients who develoepd ESRD also have a higher 5-fold mortality after cardiac surgery.

### Association of AKI and ESRD

Several epidemiological studies have reported an association between AKI and ESRD [16, 17]. In a cohort of patients who underwent coronary angiography and developed AKI, the risk of ESRD was more than 4-fold higher in patients with AKIN stage 1 and almost 12-fold higher in patients with AKIN stage 2–3 compared to patients without AKI; and the incidence of ESRD was 0.6% for that study population [16]. In another cohort of patients who underwent CABG during a 9-year period, 0.4% of the cohort developed ESRD during a mean follow-up of 4.3 ± 2.4 years [17]. In contrast, during a mean follow-up of 4.4 ± 2.8 years in our population, a total of 24 patients (0.9%) developed ESRD, which is twice as high.

It was previously thought that there is no long-term sequelae of an episode of AKI with expectant recovery of renal function. However, recent evidence from multiple observational studies demonstrated a strong reproducible association between AKI, subsequent CKD and ESRD [8, 9, 11, 16]. In some cases, this decline in renal function after AKI follows a rapid trajectory with the patient developing accelerated ESRD requiring long-term renal replacement therapy [17, 18]. We have previously reported that up to 10% of patients who developed AKI in the post-operative period after cardiac surgery have elevated serum creatinine at the point of hospital discharge [19]. It is clear that some of these patients develop accelerated ESRD within 5 years as shown in this study.

AKI is directly associated with the progression of CKD but it is also postulated that it directly causes CKD [20]. Our study confirmed a dose-dependent relationship between the increased severity of AKI and increased incidence of ESRD. Other studies have shown that multiple episodes of AKI predict the development of CKD [21]; and CKD has also been reported in children who had AKI but had no other risk factors such as hypertension, diabetes or cardiovascular disease [22]. The evidence for primary pathogenic links between AKI and CKD remains to be elucidated, but there is some suggestion that in acute tubular necrosis there is rapid progression to ESRD even in patients without pre-existing CKD [20].

These findings suggest that an episode of AKI is particularly detrimental in a cohort of cardiac surgical patients who have pre-existing risk factors for CKD. An episode of AKI in itself can cause CKD as well as worsen pre-existing CKD. Animal studies have outlined a number of causal pathways in the progression of renal dysfunction following AKI [23]. These include maladaptive repair, disordered regeneration or both [23]. However, relatively little is known about the long-term course of AKI in the setting of cardiac surgery with its attendant ischemic injuries and inflammation and these need to be addressed.

### Risk factors of AKI and ESRD

The patients who developed ESRD after AKI are mainly males with an average age of 57.3 ± 9.4 years. This is relatively young as compared to the age of initiation of dialysis in other populations, such as that in the United States where the average age of patients starting on dialysis is 64.7 ± 14.5 years [24]. This also reflects the overall younger age of presentation of heart disease in the Asian population, but the early age of presentation of ESRD is particularly alarming given the long-term morbidity and health burden of this chronic disease.

Diabetes mellitus is especially prevalent in the Asian population and diabetics who developed AKI are particularly at risk of developing ESRD. Numerous studies have shown that South Asians have decreased sensitivity to insulin when compared with other ethnic groups which may be related to increased levels of truncal fat and dysfunctional adipose tissue resulting in a blunted response to insulin [25]. It is well known that diabetes mellitus is the leading cause of ESRD worldwide [26] and the common consensus is an expected, linear, progressive and time-dependent decline of CKD to ESRD in the diabetic patient [17, 18]. Perhaps less known is that after an episode of AKI, the trajectory of accelerated ESRD is rapid, and we showed that in our population the rate of decline is as short as 5 years following cardiac surgery. This corroborates with recent publications [17, 18] and has tremendous implications on ESRD care planning as it affects mainly males of a relatively younger age group compared to their Western counterpart.

Equally important is the modification of risk factors that will retard the risk of AKI and subsequent ESRD. Patients who developed AKI and subsequent ESRD have a lower perioperative haemoglobin and higher rates of transfusion. We have shown previously that pre-operative anaemia increased the risk of AKI by 23% and Asians with a smaller body size are particularly vulnerable to the haemodilution associated with cardiopulmonary bypass and need for subsequent blood transfusion – a dual insult that exacerbates the hypoxic insult of low haemoglobin and aggravated by initiation of the systemic inflammatory response associated with blood transfusion [1]. There is debate on the optimal haemoglobin to maintain during cardiopulmonary bypass and it may well differ between diabetic and non-diabetic patients and warrants further investigation.

Pre-operative CKD is a major determinant of post-operative AKI [7]. The transient loss of renal function (i.e., AKI) in patients with pre-existing CKD can occur via a few pathways, such as the failure of renal autoregulation, abnormal vasodilatation of the renal vasculature, increased susceptibility to side effects of anti-hypertensive agents [20]. However, it is apparent that even though 29% of our cohort developed AKI, 62.9% of patients with pre-existing renal impairment had non-recovery of acute-on-chronic kidney injury with a steep downhill trajectory and accelerated ESRD [17, 18].

### Continuum of AKI, CKD, ESRD and mortality

Overall, patients who developed ESRD within the follow-up period have a 5-fold increased risk of mortality compared with those without ESRD.

It is known that patients who survive an episode of AKI are at risk for major adverse cardiovascular events as well as progression to CKD, regardless of whether there is underlying cardiovascular disease [16]. In this cardiac surgical population with underlying cardiac disease, the added insult of AKI and CKD will further aggravate cardiac disease despite successful revascularization. Studies suggest that “organ crosstalk” may be active long after the initial injury and the acute cardiorenal injury induces a vicious cycle that persists long after the acute event [27]. Pre-clinical models demonstrate that AKI can result in distant organ effects including cardiac cell apoptosis and cardiac leucocyte infiltration [28–30].

### Strengths and limitations

This is a prospective study of a cohort of cardiac surgical patients with clearly defined data points. The patient population is relatively homogenous with easy access to healthcare. The present study was protocol driven with uniform practice among the various anaesthetists, perfusionists and surgeons ensuring reliability of data.

The Singapore Renal Registry is a well-defined nationwide cohort with extensive baseline information. The data capture for ESRD is estimated to be 95% complete [15] as the National Registry of Diseases Act in 2009 mandates all physicians to notify, within 3 months from the data of diagnosis, all ESRD cases to the Singapore Renal Registry of the National Registry of Diseases Office [31].

One of the limitations is that pre-operative renal screening was done using serum creatinine which is the standard of care in Singapore and many parts of the world. Proteinuria is currently not routinely screened in patients undergoing cardiac surgery and this would be part of future prospective studies to enable better renal risk stratification and counselling.

Also, as AKI was previously believed to be self-limiting, patients were not routinely followed up at regular intervals by renal physicians for progression of renal disease.

## Conclusions

We have shown that AKI is associated with the long-term development of ESRD after cardiac surgery. The trajectory of progression from AKI to CKD and ESRD is rapid within 5 years after cardiac surgery. ESRD occurs in a younger age group in our population and leads to significant disease burden and high healthcare costs and mortality. A concerted follow-up assessment by nephrologists after hospital discharge with periodic assessment of renal function and urinary protein-creatinine ratio to assess prognosis and outcome after discharge is advocated for patients who develop AKI after cardiac surgery.

## Abbreviations

AKI: Acute kidney injury
AKIN: Acute kidney injury network
C.I.: Confidence interval
CKD: Chronic kidney disease
eGFR: Estimated glomerular filtration rate
ESRD: End-stage renal disease
HR: Hazard ratio
SORO-ESRD: Syndrome of rapid onset end-stage renal disease
